# Recurrent Cerebral Embolic Infarcts in a Patient With a Mechanical Valve: A Rare Case of Infective Endocarditis Caused by Parvimonas micra

**DOI:** 10.7759/cureus.71521

**Published:** 2024-10-15

**Authors:** Mudasar Aziz, Katherine Hartley, Dinesh Chadha

**Affiliations:** 1 Stroke, Doncaster Royal Infirmary, Doncaster, GBR

**Keywords:** acute ischemic infarct, anticoagulation, infective endocarditis, parvimonas micra, stroke

## Abstract

We present the case of a 60-year-old man with a history of mechanical mitral valve replacement and atrial fibrillation who developed infective endocarditis (IE) caused by *Parvimonas micra* (*P. micra*), resulting in recurrent cerebral infarctions. Despite increasing the intensity of anticoagulation therapy with warfarin and adding aspirin, the patient experienced four embolic cerebral infarctions. Following a single fever spike, blood cultures identified *P. micra*, although no other systemic features of IE were present. A transesophageal echocardiogram (TEE) demonstrated vegetation on the mitral valve, and a dental assessment led to the extraction of two retained roots. The patient was treated with intravenous benzylpenicillin for six weeks, followed by oral amoxicillin, totaling three months of antibiotic therapy. This case highlights a rare cause of IE which is *P. micra*, and emphasizes the importance of considering IE in patients with recurrent cerebral infarctions, particularly those with prosthetic cardiac valves.

## Introduction

Infective endocarditis (IE) is associated with high morbidity and mortality, particularly when caused by anaerobic bacteria [1]. Although anaerobic organisms are responsible for a minority of IE cases, there is an increased risk of severe complications, including thromboembolic events, which can occur in up to 50% of cases. The prompt initiation of antimicrobial therapy is crucial in reducing IE sequelae [2]. Among the anaerobic bacteria, *Parvimonas micra* (*P. micra*) is a rare but increasingly recognized pathogen in IE, particularly in those with prosthetic cardiac valves. Mainly associated with the oral flora and infections of the periodontal space, *P. micra* has been increasingly recognized as a primary pathogen in invasive infections such as prosthetic joint infections, pericarditis, and meningitis, with further complications once IE occurs. [3]. This paper presents a peculiar case of *P. micra* IE that mainly manifested through multiple cerebral embolic infarcts, thereby emphasizing the need for further evaluation of IE among patients with repeated cerebral events, especially if other risk factors, including prosthetic valves, are present.

## Case presentation

A 60-year-old male with a history of atrial fibrillation and mechanical mitral valve replacement for severe mitral regurgitation presented with recurrent episodes of cerebral infarction. Three years after valve replacement surgery, he experienced a left cerebellar infarct, presenting with a headache, dizziness, blurred vision, and a sense of imbalance. Magnetic resonance imaging confirmed the infarct. Five months later, he returned with dizziness, nausea, and dysarthria; imaging revealed multiple embolic infarcts in the right cerebellar, left periventricular, and parietal regions. Two weeks after that, he developed new symptoms of weakness and visual changes, leading to a diagnosis of left hemianopia and left hemiparesis, with fresh infarcts seen in the right temporal and occipital regions on MRI.

**Figure 1:**
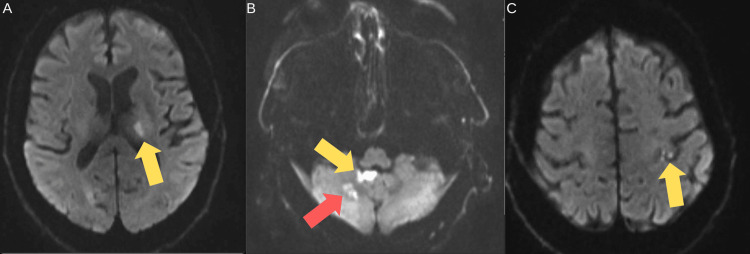
Magnetic resonance imaging of the head sequences showing recurrent infarcts Diffusion-weighted images show infarcts in the left thalamus (A), right cerebellar hemisphere (red arrow), vermis (yellow arrow) (B), and left frontal cortex (C).

Due to these recurrent events, the patient's international normalized ratio (INR) target was increased to 3.0, and aspirin was added to his regimen. A transthoracic echocardiogram (TTE) was inconclusive due to poor image quality but suggested that the mitral valve was well-seated and functioning normally. Four months later, he presented with right arm and leg weakness, with an MRI scan revealing infarcts in the left thalamus, right cerebellar hemisphere and vermis, and the left frontal cortex (Figure 1). Within 24 hours of admission, he developed a fever of 38.6°C.

Laboratory investigations showed persistently elevated C-reactive protein (CRP) and erythrocyte sedimentation rate (ESR) levels, which had been raised since the first stroke (Table 1). On admission for the fourth stroke, the CRP was 37 mg/L, peaking at 63 mg/L two days after the fever onset. The white cell count (WCC) was elevated at 16.7 × 10⁹/L, consistent with previous episodes. The ESR levels during the stroke episodes ranged from 30 to 40 mm/hour.

**Table 1:** The patient's laboratory blood tests during ischemic and notable events CRP: C-reactive protein; WCC: white cell count; ESR: erythrocyte sedimentation rate

	Reference range	Baseline	First stroke	Second stroke	Third stroke	Fourth stroke	Six weeks of treatment	Three months of treatment
CRP	0.0-5.0 mg/L	9.4	21.2	26	19.4	37.6	27.3	2.5
WCC	4.0 - 11.0 x 10⁹/L	6.8	17.2	14.8	13.4	16.7	8.3	9.1
Neutrophils	2.0 - 7.5 x 10⁹/L	4.2	13.6	12.1	10.6	13.9	2.8	5.3
ESR	1.0 - 15.0 mm/hr	15	40	35	30	39	Not available	21

Despite the absence of localized infection symptoms, blood cultures taken during the fever spike were positive for *P. micra*, confirmed by six subsequent cultures. A CT scan of the thorax, abdomen, and pelvis did not reveal additional infection sources. However, a TEE demonstrated a 9-mm vegetation on the mitral valve without peripheral stigmata of IE.

Given the patient’s risk factors for IE, including poor dentition and a mechanical mitral valve, an orthopantomogram was performed, showing no dental caries or abscesses. However, a maxillofacial review identified two retained roots in the upper incisor area, which, despite not showing signs of infection, were considered potential sources. A repeat TEE after four weeks of antibiotic therapy showed the vegetation had become less prominent. The patient was treated with intravenous benzylpenicillin (1,200 mg every four hours) for six weeks, based on sensitivity testing. Dental extraction of the two retained roots was performed towards the end of the treatment. Following the intravenous therapy, the patient received oral amoxicillin (1 g three times daily), completing a three-month antibiotic course, and ensuring appropriate management as guided by the microbiology team's assessment. Repeat blood cultures were negative, and surgery was deemed unnecessary by the IE multidisciplinary team.

## Discussion

*Parvimonas micra* is an anaerobic Gram-positive coccus, previously known as *Peptostreptococcus micros *(*P. micros*), but it was reclassified in 2006 under the *Micromonas *genus [4]. It is part of the normal oropharyngeal and gastrointestinal flora and is commonly associated with periodontal infections. However, it is increasingly recognized as a cause of invasive infections, including IE [3].

The first reported case of *P. micra* IE was published in 2015, involving native aortic and mitral valve involvement, requiring valve replacement and an ascending aorta graft [3]. *Parvimonas micra* has also been reported in cases of pacemaker-associated IE, requiring device removal until the infection was cleared with antimicrobials [5].

A systematic review of *P. micra* found four cases of IE, all associated with a history of dental extraction [6]. Additionally, a case series of IE included a patient who presented with chest pain, shortness of breath, fever, and malaise and was found to have *P. micra*-associated IE of the native mitral valve, leading to embolic cerebral infarcts [7].

Given the change in terminology, we also reviewed publications involving *P. micros* IE, which has been reported in the context of congenital subvalvular stenosis, cerebral cavernous malformation, and lumbar discitis [8-10].

To the best of our knowledge, this is the first documented case in which *P. micra*-associated IE presented with recurrent cerebral embolic infarcts in a patient with a mechanical heart valve. While atrial fibrillation and mechanical heart valves are known risk factors for embolic stroke, it is vital clinicians maintain a high index of suspicion of IE if patients experience recurrent embolic stroke events despite adequate antithrombotic treatment. In addition, it is apparent that IE may not present with classical systemic features or peripheral stigmata, especially when caused by bacteria such as *P. micra*. This case emphasizes the need to establish an accurate diagnostic strategy, consisting of repeated blood cultures and detailed echocardiographic assessment, even when classical investigative procedures do not align with the clinical presentation. Furthermore, meticulous dental evaluation is important, even in the absence of infection-related manifestations, as eliminating potential oral foci reduces the risk of relapse and further strokes.

## Conclusions

Recurrent strokes despite anticoagulation should prompt a search for IE. This case highlights that potential pathogens, such as *Parvimonas micra*, may cause IE in the absence of classical systemic features of the disease. The administration of timely and effective antimicrobial therapy, along with multi-specialized management, is imperative for preventing progression to serious complications and ensuring favorable outcomes in such cases.
